# Regulome-based characterization of drug activity across the human diseasome

**DOI:** 10.1038/s41540-022-00255-4

**Published:** 2022-11-07

**Authors:** Michio Iwata, Keisuke Kosai, Yuya Ono, Shinya Oki, Koshi Mimori, Yoshihiro Yamanishi

**Affiliations:** 1 Department of Bioscience and Bioinformatics, Faculty of Computer Science and Systems Engineering, Kyushu Institute of Technology, Iizuka, Fukuoka 820-8502 Japan; 2 Department of Surgery, Kyushu University Beppu Hospital, Beppu, Oita 874-0838 Japan; 3 Department of Drug Discovery Medicine, Kyoto University Graduate School of Medicine, Sakyo-ku, Kyoto 606-8507 Japan; 4 Precursory Research for Embryonic Science and Technology, Japan Science and Technology Agency, Kawaguchi, Saitama 332-0012 Japan

**Keywords:** Regulatory networks, Drug discovery and development

## Abstract

Drugs are expected to recover the cell system away from the impaired state to normalcy through disease treatment. However, the understanding of gene regulatory machinery underlying drug activity or disease pathogenesis is far from complete. Here, we perform large-scale regulome analysis for various diseases in terms of gene regulatory machinery. Transcriptome signatures were converted into regulome signatures of transcription factors by integrating publicly available ChIP-seq data. Regulome-based correlations between diseases and their approved drugs were much clearer than the transcriptome-based correlations. For example, an inverse correlation was observed for cancers, whereas a positive correlation was observed for immune system diseases. After demonstrating the usefulness of the regulome-based drug discovery method in terms of accuracy and applicability, we predicted new drugs for nonsmall cell lung cancer and validated the anticancer activity in vitro. The proposed method is useful for understanding disease–disease relationships and drug discovery.

## Introduction

Diseases are caused by dysfunctions in human biological systems consisting of genes, proteins, and pathways. Disease pathogenesis is generally considered as disease-specific; however, characteristic molecular features are often similar among different diseases, suggesting a commonality in underlying molecular mechanisms^1–3^. Disease states are characterized by impaired expression of genes; thus, the commonalities shared among diseases could be explained by certain gene expression patterns. Drugs are expected to recover the gene expression system away from the impaired state to normalcy through disease treatment, with the implication that appropriate drugs should cancel disease-specific gene expression patterns.

Drug repositioning, namely, the identification of new therapeutic indications (i.e., applicable diseases) of existing drugs, is an efficient drug discovery strategy^4–7^. A popular computational approach for drug repositioning is the usage of chemically-induced gene expression profiles stored in the Connectivity Map (CMap)^8^ and the Library of Integrated Network-based Cellular Signatures (LINCS)^9^. The prediction assumes that gene expression patterns perturbed by treatment with a drug are inversely correlated with those of a disease of interest if the drug applies to the disease^10–12^. This assumption was established based on the observation of significant inverse correlations between gene expression patterns of Alzheimer’s disease and those upon the administration of drugs for treating Alzheimer’s disease^8^. Several algorithms have been developed for the inverse correlation-based drug repositioning approach^13,14^ to associate chemically-induced transcriptome data with diseases. However, the understanding of gene regulatory machinery underlying drug activity or disease pathogenesis is far from complete.

Gene expression is regulated by transcription factors in a coordinated manner^15^; therefore, each transcription factor could be a drug(s) target for treating various diseases^16^. To evaluate the intracellular activity of drugs, the activity of transcription factors was previously assessed using a multiplex reporter system^17^. Recent sequencing technologies (e.g., ChIP-seq) have enabled comprehensive characterization of associations between genes and transcription factors. Thus, a comprehensive analysis of ChIP-seq data is expected to be used for deciphering gene regulatory machinery. Alternatively, the method of predicting gene regulatory network comprising of genes and their regulators (i.e., transcription factors) by focusing on the binding motif sequences has been proposed (SCINIC^18^). A data-mining platform, ChIP-Atlas^19,20^, has been developed by fully integrating public ChIP-seq data with an established protocol. ChIP-Atlas enables the analysis of given genomic intervals using global protein–DNA binding data. For example, for disease-specific differentially expressed genes, highly-enriched transcription factors can be considered as regulators of disease-specific gene expression. Thus, gene expression can be explained at the regulome level, which provides a new perspective on disease states.

In this study, we perform large-scale regulome analysis for various diseases in terms of gene regulatory machinery. Transcriptome signatures were converted into regulome signatures of transcription factors by fully integrating all possible ChIP-seq data. Our comprehensive examination shows that regulome-based correlations between diseases and their approved drugs are much clearer than the transcriptome-based correlations. We proposed a regulome-based method for drug discovery and demonstrated the usefulness of the proposed method in terms of accuracy and applicability. We predicted new drugs for nonsmall cell lung cancer using the regulome data only and validated the anticancer activity in vitro. The proposed method enabled clarification of cell systems in terms of gene regulatory machinery, which could lead to understanding disease−disease relationships and drug discovery.

## Results

### Overview of the proposed method

**Fig. 1:**
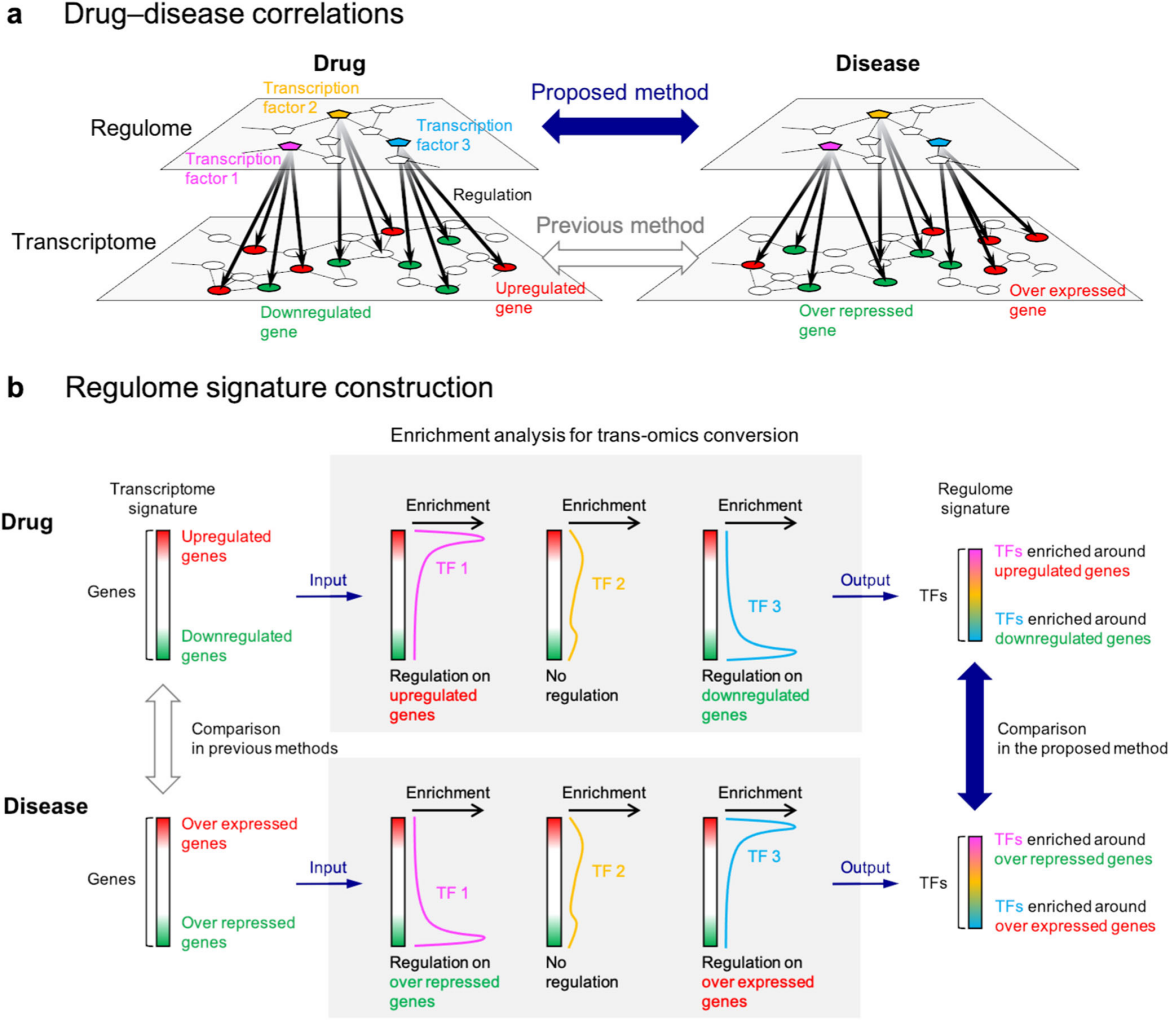
Overview of the proposed method. **a** Evaluation of drug−disease correlations at the different omics level. Circles in the transcriptome layer denote genes and pentagons in the regulome layer denotes transcription factors. **b** Conversion of transcriptome signatures into regulome signatures. Drug-induced and disease-specific transcriptome signatures are converted into regulome signatures. Up- and downregulated genes in drug-induced transcriptome signatures and over expressed and repressed genes in disease-specific transcriptome signatures are manually extracted (left). Enrichment of transcription factors on the regulated genes are statistically evaluated on the ChIP-Atlas platform (center). The resulting FDR-corrected *p*-values are used for constructing regulome signatures (right).

In this study, we evaluate drug−disease correlations at the regulome level for predicting drug candidates for various diseases. Gene regulations in cell systems are reflected by transcriptome and regulome data (Fig. 1a). Transcriptome data contains information on genes and regulome data contains information on their regulators (i.e., transcription factors). In previous methods, drug−disease correlations have been evaluated at the transcriptome level^10,12,21–23^, whereas, in the proposed method, the correlations are evaluated at the regulome level. Thus, it can be expected that the new correlations between drugs and diseases are clarified.

We converted transcriptome signatures into regulome signatures by evaluating the enrichment of transcription factors on regulated genes (Fig. 1b). First, transcriptome signatures were curated from public repositories. Drug-induced transcriptome signatures were obtained from the LINCS program^9^. Disease-specific transcriptome signatures were obtained from the CRowd Extracted Expression of Differential Signatures (CREEDS) database^24^ and The Cancer Genome Atlas (TCGA) program (https://www.cancer.gov/tcga). Second, for drugs, up- and downregulated genes in each drug-induced transcriptome signature were extracted. The enrichment of each transcription factor on a set of regulated genes was statistically evaluated using Fisher’s exact probability test. Finally, regulome signatures were constructed using FDR-corrected p-value given for each transcription factor; where transcription factors enriched around up- and downregulated genes were scored by positive and negative values, respectively. Similarly for diseases, transcription factors enriched around over expressed and repressed genes in disease-specific transcriptome signatures were scored by positive and negative values, respectively.

### Regulome-based characterization of diseases

**Fig. 2:**
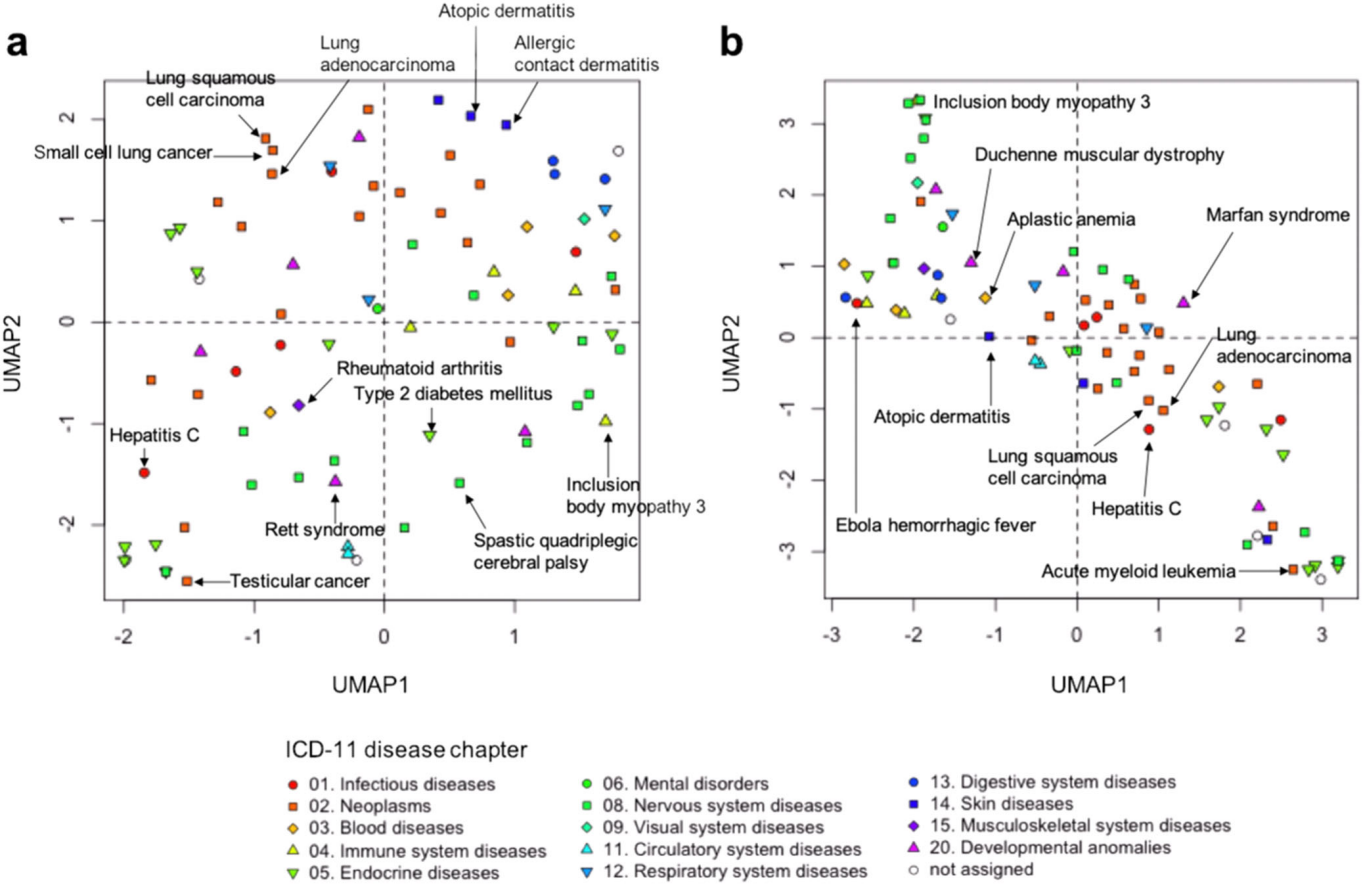
Disease–disease relationships in terms of transcriptome and regulome signatures. **a** Scatter plots of diseases obtained after applying Uniform Manifold Approximation and Projection (UMAP) to transcriptome-based disease signatures. **b** Scatter plots of diseases obtained after applying UMAP to regulome-based disease signatures. Disease symbols are colored according to the ICD-11 disease chapters.

To identify the commonality of different diseases, we applied Uniform Manifold Approximation and Projection^25^ on a set of disease signatures. When using disease-specific transcriptome signatures, diseases that have similar gene expression patterns were closely located (Fig. 2a and Supplementary Fig. 1a). For example, “atopic dermatitis” and “allergic contact dermatitis” were closely located, as they belong to chapter 14 (diseases of the skin) that is defined in the 11th revision of the International Classification of Diseases (ICD-11)^26^, implying that diseases of the same organ tend to be similar in terms of gene expression patterns and the transcription signatures from similar cell types could be closely associated. However, there are many exceptional cases of closely clustered diseases in different ICD chapters and those within the same ICD chapter were clustered separately. Therefore, transcriptome-based disease classification was different from organ-based disease classification in traditional medicine. When using regulome signatures, diseases that are regulated by similar transcription factors were closely located (Fig. 2b and Supplementary Fig. 1b). For example, almost all cancers were closely located, as they belong to chapter 02 (neoplasms). These diseases were separately located in the case of transcriptome signatures, suggesting that regulome-based disease classification differs from transcriptome-based disease classification.

### Regulome signatures reveal distinct correlations between diseases and their approved drugs

Generally, a drug-induced transcriptome signature is considered to be inversely correlated with a disease-specific transcriptome signature if the drug is effective for treatment of the disease^10,12,21–23^. Thus, we investigated whether disease-specific transcriptome signatures exhibited clear inverse correlations with drug-induced transcriptome signatures of known drugs approved for the corresponding diseases.

**Fig. 3:**
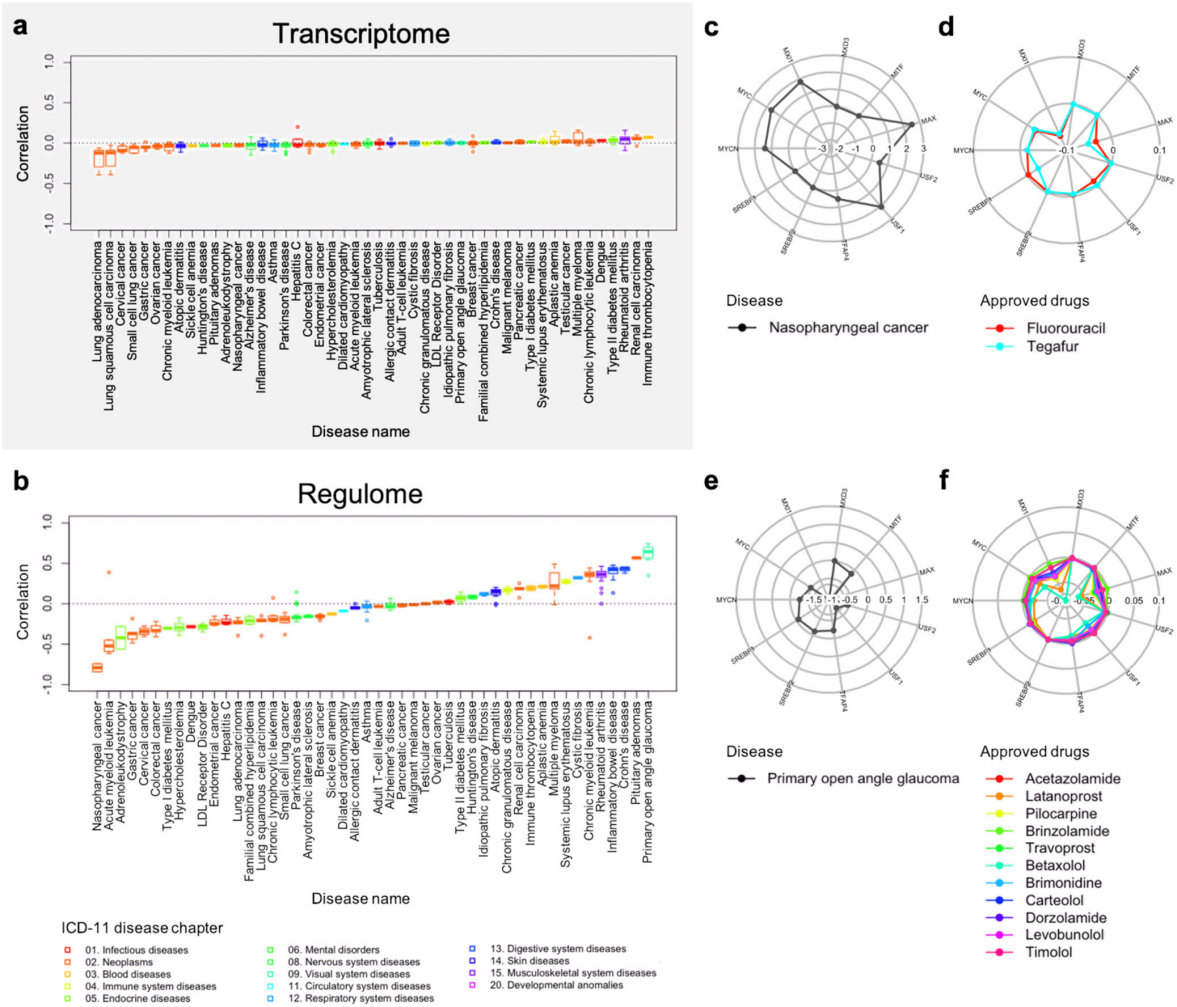
Relationships between diseases and their approved drugs. **a** Distribution of correlation scores between transcriptome-based drug and disease signatures. **b** Distribution of correlation scores between regulome-based drug and disease signatures. Each boxplot represents correlation scores of the drugs approved for the corresponding disease. The horizontal axis indicates the list of diseases and the vertical axis indicates cosine correlation coefficient. Diseases are listed in order of increasing median correlation scores. In the box plots: center line, median; box, interquartile range; whiskers, 1.5× interquartile range; dots, outliers. **c** Radial plot of enrichment scores for transcription factors in the regulome signature of nasopharyngeal cancer. **d** Radial plot of enrichment scores for transcription factors in the regulome signature of approved drugs for nasopharyngeal cancer. **e** Radial plot of enrichment scores for transcription factors in the regulome signature of primary open angle glaucoma. **f** Radial plot of enrichment scores for transcription factors in the regulome signature of approved drugs for primary open angle glaucoma.

We analyzed known drug–disease associations involving 48 diseases where at least one drug was approved for the corresponding disease. When comparing transcriptome signatures, inverse correlations were not always observed (Fig. 3a), suggesting that the inverse correlation-based method might not always work in practice. In contrast, when comparing regulome signatures, clear correlations were observed for many diseases (Fig. 3b). An inverse correlation between the two signatures was observed for cancers such as “nasopharyngeal cancer”, “acute myeloid leukemia”, and “gastric cancer”, whereas a positive correlation was observed for immune system diseases such as “Crohn’s disease”, “inflammatory bowel disease”, and “rheumatoid arthritis”. The strongest inverse correlation was observed for “nasopharyngeal cancer”, where *MYC* (*MYC* proto-oncogene, bHLH transcription factor), *MAX* (*MYC* associated factor X), and *MXI1* (*MAX* interactor 1, dimerization protein) have positive enrichment scores in the disease-specific regulome signature (Fig. 3c and Supplementary Fig. 2) and these transcription factors have negative enrichment scores in the regulome signatures of approved drugs for the disease (Fig. 3d and Supplementary Fig. 3). The Pearson’s correlation coefficient scores were from −0.935 to −0.502 for each category of transcription factors, except for the transcription factors in the “Other basic domain” category, the correlations were significant (*P* < 0.05; Supplementary Fig. 4). In contrast, the strongest positive correlation was observed for “primary open angle glaucoma”, where these transcription factors have negative enrichment scores in both the disease-specific regulome signature (Fig. 3e and Supplementary Fig. 5) and the regulome signatures of approved drugs for the disease (Fig. 3f and Supplementary Fig. 6). The Pearson’s correlation coefficient scores were from 0.467 to 0.873 for each category of transcription factors, and except for the “Other basic domain” and “Other transcription factors” categories, the correlations were significant (*P* < 0.05; Supplementary Fig. 7). These results suggest that regulome signatures contribute to clear correlations between drugs and diseases and the correlations are different among the types of diseases.

### Performance evaluation

In this study, we applied the proposed method to the prediction of candidate drugs for various diseases. The diseases and their approved drugs may not always have inverse correlations; thus, we predicted candidate drugs for each disease by considering the positive and negative correlations separately. If the disease was positively correlated with its approved drugs, drugs that have positive correlations were predicted with high prediction scores as candidate drugs for the disease. In contrast, if the disease was inversely correlated with its approved drugs, drugs that have negative correlations were predicted with high prediction scores as candidate drugs for the disease.

**Fig. 4:**
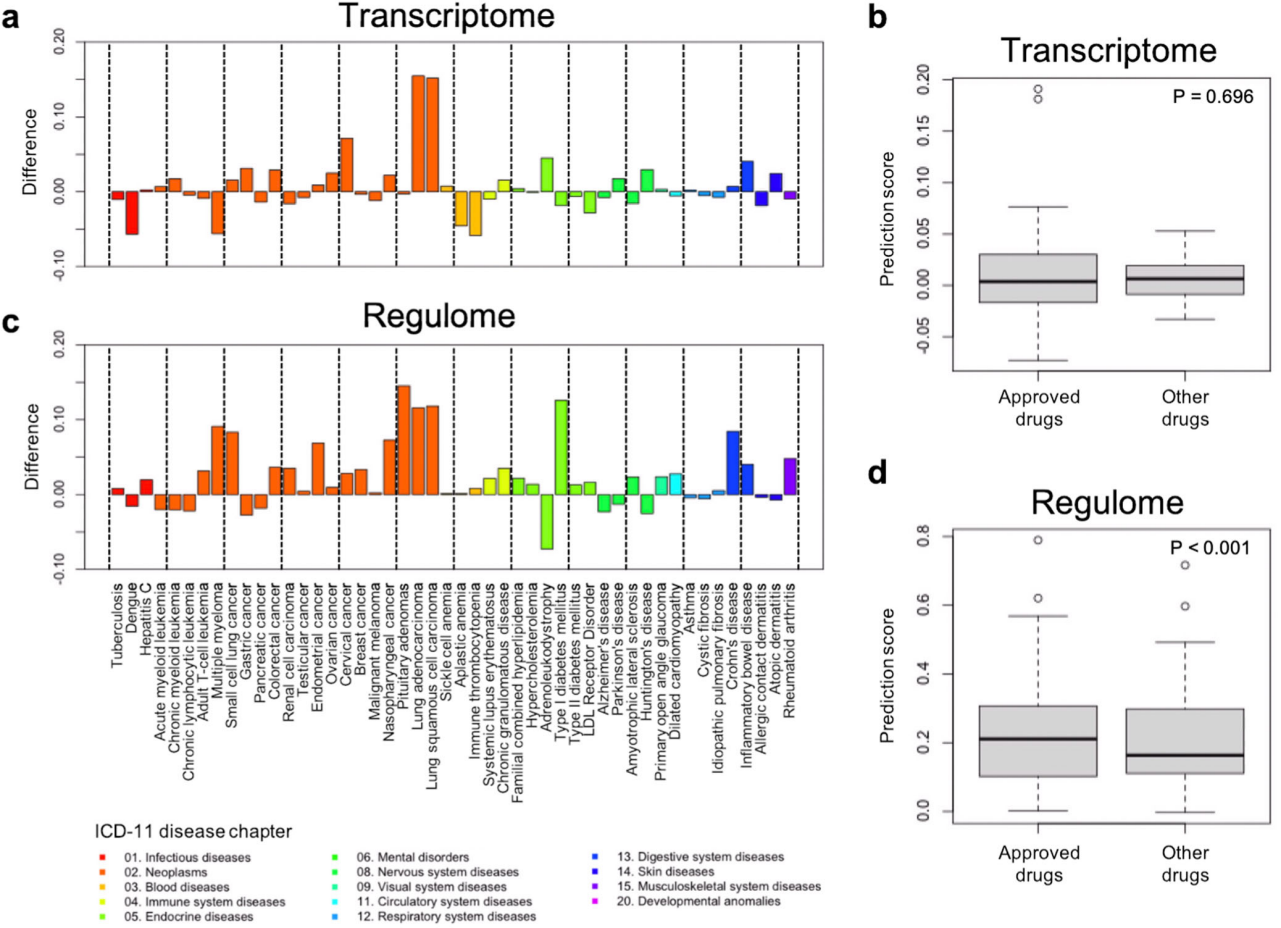
Performance evaluation for drug indication prediction. **a** Difference between the prediction scores for approved drugs and those for other drugs by the transcriptome-based prediction. **b** Distribution of prediction scores for approved and other drugs by the transcriptome-based prediction. **c** Difference between the prediction scores for approved drugs and those for other drugs by the regulome-based prediction. **d** Distribution of prediction scores for approved and other drugs by the regulome-based prediction. The positive difference means the prediction scores for approved drugs are larger than those for other drugs. Bars are colored according to the ICD-11 disease chapters. All *P*-values were determined by the Wilcoxon signed-rank test. In the box plots: center line, median; box, interquartile range; whiskers, 1.5× interquartile range; dots, outliers.

We compared the prediction scores between approved drugs for each disease and other drugs in transcriptome-based and regulome-based predictions. For example, in the transcriptome-based prediction, approved drugs for “adrenoleukodystrophy” have higher prediction scores than other drugs (Fig. 4a), implying that the transcriptome-based method can predict drug candidates for the disease. However, it is not always the case that the approved drugs have higher scores than other drugs. Overall, there is no significant difference in prediction scores between approved and other drugs (Fig. 4b; *P* = 0.696, Wilcoxon signed-rank test). In contrast, in the regulome-based prediction, approved drugs for almost all diseases have higher prediction scores (Fig. 4c). Also, there are several diseases; e.g., “multiple myeloma”, “Type I diabetes mellitus”, and “rheumatoid arthritis”, for which the regulome-based method can predict approved drugs with higher prediction scores. Overall, there is a significant difference in prediction scores between approved and other drugs (Fig. 4d; *P* < 0.001). These results suggest that the use of regulome data for predicting drug indications has a potential to enhance the performance of the existing transcriptome-based prediction (see Supplementary notes and Supplementary Figs. 8 and 9).

### Anticancer activity of Danazol was validated using in vitro experiments

**Fig. 5:**
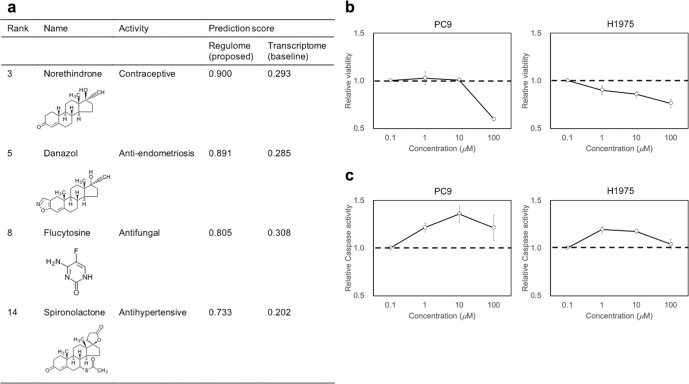
Experimental validation of predicted anticancer effects of drugs. **a** List of tested drugs and their chemical structures. **b** The effect of an anti-endometriosis drug, danazol, on the viability of nonsmall cell lung cell lines. **c** The effect of danazol on the apoptosis induction. Cell lines were treated with various concentrations of danazol. The horizontal axis represents the concentration on a logarithmic scale. The vertical axis represents the relative viability and relative apoptosis induction (caspase activity). Plot shows means and standard deviations for triplicate experiments.

To experimentally validate the efficacy of predicted drugs, we tested the anticancer activity of some drugs on nonsmall cell lung cancer. From the high-scoring drugs, we selected commercially available drugs. Taking into consideration budget constraints, we chose the 4 nonanticancer drugs: Norethindrone (contraceptive), Danazol (anti-endometriosis), flucytosine (antifungal), and spironolactone (antihypertensive) for experimental validation (Fig. 5a). Except for flucytosine, known targets of these test drugs are transcription factors. PGR (progesterone receptor) is the target for norethindrone. The targets of danazol are AR (androgen receptor), PGR, and ESR1 (estrogen receptor 1). The target of flucytosine is TYMS (thymidylate synthetase). Spironolactone targets NR3C2 (nuclear receptor subfamily 3 group C member 2). It is worth noting that high scores were not achieved using transcriptome signatures.

We tested for anticancer activity regarding cell viability and apoptosis. To evaluate the effect of the selected drugs, we used human nonsmall cell lung cancer cell lines: H1975, PC9, H3122, and HC827. Among the tested drugs, we could observe the anticancer activity of danazol. In the cell viability assay, Danazol induced a dose-dependent decrease in the viability of PC9 and H1975 (Fig. 5b). Also, in the apoptosis assay, Danazol showed a dose-dependent upregulation of apoptosis (Fig. 5c). Two of three known targets, AR and ESR1, had positive enrichment scores (0.002 and 0.001, respectively), implying that these transcription factors were enriched around danazol-induced regulated genes. In fact, it is reported that steroid hormones, including androgen and estrogen, have a crucial role in the progression of non-small cell lung cancer^27,28^. Therefore, danazol could have anticancer activity by regulating steroid hormones. Danazol has recently been reported to have anticancer activity^29^. In multidrug-resistant cancer cells, Danazol has been shown to induce cytotoxicity, cell cycle arrest, and apoptosis^30^. Danazol also can dysregulate the cell cycle and induce apoptosis in breast cancer cells^31^. Thus, the predicted drug may be a promising candidate for treating nonsmall cell lung cancer (see Supplementary notes and Supplementary Fig. 10).

## Discussion

In this study, we present a computational method to predict drug candidates for various diseases by focusing on gene regulatory machinery. The originality of the proposed method lies in the construction of regulome signatures representing the enrichment of transcription factors involved in the differential gene expressions. Our analysis revealed that the regulome-based classification of diseases uncovers new disease associations not apparent with transcriptome-based analysis. Also, we identified that diseases and their approved drugs have clear correlations in regulome signatures, and the correlation is useful for predicting drug candidates of diseases. We predicted new drugs for nonsmall cell lung cancer using the regulome data only and validated the anticancer activity in vitro. The proposed method enables us to clarify cell systems in terms of gene regulatory machinery for drug discovery.

There are several frameworks to drug discovery. The target-based framework is popular, where the search for compounds interacting with a specific target molecule or pathway (e.g., apoptosis) is performed. Another possibility is the system-based framework. Following the compound−target interaction, many genes/pathways in the cellular system are regulated in a complicated manner; therefore, it is desirable to consider the behavior of all genes/pathways in the cellular system to have a better insight into the mode of action of compounds. In the context of the system-based framework, the hypothesis that drugs are to return the transcriptome/regulome to normalcy is expected to be helpful. In fact, based on the assumption, many drug candidate compounds were discovered for various diseases, including Alzheimer’s disease^8^, prostate cancer^11^, and metastatic colorectal cancer^12^.

One disadvantage of the transcriptome-based approach is that individual gene expression patterns are very varied; therefore, detectable disease–drug correlation is weak, as shown in the analysis of disease−approved drug correlations (Fig. 3a). Thus, finding common correlation patterns for various diseases belonging to the same disease class is difficult. In contrast, in the regulome-based approach, the expression patterns of different genes can sometimes be explained by the same transcription factor: therefore, detectable disease−drug correlation is relatively strong (Fig. 3b). Thus, it is easier to find common correlation patterns for different diseases belonging to the same disease class (e.g., a negative correlation for cancer and a positive correlation for immune system diseases). The regulome-based approach may contribute to a better insight into the drug-induced gene expression machinery for each disease class from the viewpoint of transcription factors compared with the transcriptome-based method. Therefore, the proposed regulome-based approach could have an advantage over the transcriptome-based approach regarding interpretability.

We constructed regulome signatures statistically from extensive ChIP-seq data. The ChIP-seq data was manually curated from several experiments and reposited in ChIP-Atlas database. Due to the difference of tissues or cell types used in each experiment, for constructing regulome-based disease signatures, we selected appropriate cell types (e.g., lung) for each disease (e.g., nonsmall cell lung cancer). This operation is considered reasonable because each cell type has a different gene regulatory system; however, it is not always the case that sufficient ChIP-seq data is available from each cell type. Thus, the constructed regulome-based disease signatures could be limited by the amount of ChIP-seq data. This limitation can be overcome in the future by a large-scale ChIP-seq experiment for various cell types.

Drug signatures are assumed to be inversely correlated with disease signatures if the drugs have therapeutic effects on the corresponding diseases. However, a previous CMap-based study reported that there were limited inverse correlations for most diseases^12^. In fact, transcriptomic inverse correlations between diseases and their approved drugs were not clearly observed in this study. In contrast, regulomic correlations were clearly observed in various diseases. For example, inverse and positive correlations were clearly observed in cancers and immune system diseases, respectively. A possible explanation for the positive correlation is that disease-specific gene expression patterns might reflect potential immunological responses in patients. The immunological responses are necessary to recover impaired biological systems, which suggests that the immune system diseases and their approved drugs have similar gene regulatory systems for treating diseases. This finding could provide a new chemotherapeutic strategy for each disease.

Many diseases lack any treatment or for which a drug profile is not available in practice. The regulome-based approach makes it easier to identify common correlation patterns for different diseases belonging to the same disease class (e.g., a negative correlation for cancer, positive correlation of immune system diseases) (Fig. 3b). Given a disease for which a drug profile is unavailable, it is possible to make predictions using the prior information on positive/negative correlations for the same disease class to which the given disease belongs.

We validated the anticancer activity on nonsmall cell lung cancer cell lines in vitro experiment. Out of four experimental drugs, danazol (anti-endometriosis) showed anticancer activity in PC9 and H1975 cell lines. The other experimental drugs, norethindrone (contraceptive), flucytosine (antifungal), and spironolactone (antihypertensive), are previously reported to have anticancer properties^32–34^; however, we did not observe their anticancer activities in nonsmall cell lung cancer cell lines. Also, PC9 harboring an EGFR exon 19 deletion mutation and HCC827 expressing an EGFR exon 19 deletion mutation showed different responses to drugs. In this study, we could not investigate the regulome difference between the two cell lines due to the lack of transcriptome profiles for the cell lines; thus, the regulome profiling of various cancer cell lines could be interesting to characterize the cell lines in more detail. Also, we constructed disease-specific regulome signatures without considering gene mutations in patients. Therefore, consideration of gene mutations enables more reliable predictions of drug candidates.

In summary, we found that the proposed regulome-based approach enables to identify new associations between drugs and diseases. Although there might be a potential to improve regulome signatures from a biological perspective, our proposed method opens a door for understanding drug activities and disease states in terms of gene regulatory machinery.

## Methods

### Drug-induced transcriptome data

In the LINCS program^9^, gene expression profiles were obtained based on the L1000 mRNA profiling assay (http://www.lincsproject.org). The gene expression profiles, namely, GSE70138 and GSE92742, were obtained from the GEO database. This dataset is based on 93 human cell lines with various cellular perturbations. The LINCS database provides drug-induced gene expression data for 978 landmark genes known as the “L1000 genes” at five levels of the data processing pipeline. The “level 5” dataset is wholly processed by collapsing replicates and comprises differential gene expression signatures. In addition, the “level 5” dataset is recommended to use in LINCS. Therefore, in this study, we used “level 5” moderated Z-scores data.

The gene expression levels were measured at 3, 6, 24, 48, and 144 h after drug treatment. Each gene expression profile (591,855 in total) was represented by a “sig_id”. We used 312,596 compound-treatment profiles (denoted as “trt_cp”) in total. For each compound, the corresponding International Chemical Identifier code (InChIKey) was also obtained from GEO.

### Disease-specific transcriptome data

Gene expression profiles with patients of various diseases were obtained from the CREEDS^24^. This database was created based on the results of a re-analysis of disease-specific gene expression data from the Gene Expression Omnibus^35^. The gene expression profiles consisted of the scores computed with the Characteristic Direction method^36^ that compared the gene expression measured in disease tissue with that measured in control tissue. The over expressed and over repressed genes had gene expression scores denoted as “up_genes” and “down_genes” fields, respectively.

We used 695 profiles annotated as “manual disease signatures”, because the profiles were assigned disease ontology IDs (DOIDs)^37^. The DOIDs were converted into their corresponding KEGG DISEASE^38^ IDs via the medical subject headings terms or the Online Mendelian Inheritance in Man (OMIM)^39^ database. We extracted the profiles obtained from humans, which resulted in 79 diseases and 14,804 genes (Supplementary Table 1). Out of 79 diseases, 46 diseases had at least one approved drug.

Gene expression profiles with patients with nonsmall cell lung cancers; i.e., lung adenocarcinoma and lung squamous cell carcinoma, were obtained from TCGA program (https://www.cancer.gov/tcga). We downloaded the transcriptome profiling data normalized by the Fragments Per Kilobase of transcript per Million mapped reads upper quartile (FPKM-UQ) method via Genomic Data Commons Data Portal (https://portal.gdc.cancer.gov) on February 22, 2021. The total number of samples was 1145 from 1016 cases of 2 cancer studies (Supplementary Table 2). To identify approved drugs for each cancer, we manually assigned the corresponding KEGG DISEASE IDs to each cancer. Note that the gene expression pattern in recurrent tumors is clinically different from that in primary tumors due to genetic variations, implying that primary and recurrent tumors should be analyzed separately. However, in this study, nearly all non-small cell lung cancer samples were obtained from primary tumors (1035 out of 1145 samples; see Supplementary Table 2 for more details). Therefore, we did not separate primary and recurrent tumors.

### Construction of transcriptome-based signatures

For drugs, we represented drug-induced transcriptome-based signatures using a feature vector:

1 $${{{\boldsymbol{x}}}}^{{{{\mathrm{trans}}}}} = \left( {x_1^{{{{\mathrm{trans}}}}},x_2^{{{{\mathrm{trans}}}}}, \cdots ,x_p^{{{{\mathrm{trans}}}}}} \right)^{{{\mathrm{T}}}},$$

where *p* is the number of genes (*p* is 978 in this study). Each element in the signature was defined as the difference between the gene expression value measured after drug treatment and that measured in the corresponding controls (the plate background). In this study, to make a fair comparison of the performance between the transcriptome-based method and the regulome-based method, we used the gene expression ratios for only the top 5% and bottom 5% genes, and those for the remaining genes were assigned a value of zero. Note that the top- and bottom-ranked genes were used for analyzing the enrichment of transcription factors as regulated genes. In addition, to handle the problem of the noise contained in gene expression values, the use of only regulated genes was proposed to remove the noise effects in many previous studies^14,40,41^.

For the diseases, we represented each disease-specific transcriptome signature using a feature vector as follows:

2 $${{{\boldsymbol{y}}}}^{{{{\mathrm{trans}}}}} = \left( {y_1^{{{{\mathrm{trans}}}}},y_2^{{{{\mathrm{trans}}}}}, \cdots ,y_q^{{{{\mathrm{trans}}}}}} \right)^{{{\mathrm{T}}}},$$

where *q* is the number of genes (*q* is 14,639 in this study). For CREEDS diseases, each element in the signature was the score defined in the CREEDS database. We averaged multiple signatures for the same disease and constructed a disease-specific transcriptome signature for each of 79 diseases. Note that the positive and negative scored genes were used for analyzing the enrichment of transcription factors as over expressed or repressed genes. For TCGA cancers, each element in the signature was log2 fold change in gene expression in tumor tissues (sample types are “01: Primary Solid Tumor” and “02: Recurrent Solid Tumor”) compared with that in normal tissues (sample type is “11: Solid Tissue Normal”). We averaged multiple signatures for the same cancer study and constructed a disease-specific transcriptome signature for each of cancers. In this study, we used the log2 fold changes only for the top 5% and bottom 5% genes, and those for the remaining genes were assigned a value of zero. The top- and bottom-ranked genes were used for analyzing the enrichment of transcription factors as over expressed or repressed genes. 848 out of 14,639 genes were in common with genes in drug-induced transcriptome signature, ${{{\boldsymbol{x}}}}^{{{{\mathrm{trans}}}}}$.

### Evaluation of enrichment of transcription factors on regulated genes

We evaluated the enrichment of transcription factors on regulated genes using the ChIP-Atlas database^19^. Top 5% genes in the drug-induced transcriptome signature (i.e., ${{{\boldsymbol{x}}}}^{{{{\mathrm{trans}}}}}$) and bottom 5% genes in the signature were assumed as up- and downregulated genes, respectively. Positive and negative scored genes in the disease-specific transcriptome signature (i.e., ${{{\boldsymbol{y}}}}^{{{{\mathrm{trans}}}}}$) were assumed as over expressed and repressed genes, respectively. We statistically evaluated the binding of transcription factors on the region between ±5000 base pairs from the transcriptional start site of each gene. Peak-caller MACS2 was used to calculate the statistical significance of the binding (−10*Log10[MACS2 Q-value]). We set the threshold for the statistical significance to 100. For drugs, to evaluate the imbalances of enrichment on up- and downregulated genes, we calculated the value of fold enrichment (FE). If the enrichment is lean to the upregulated genes, the value of FE takes more than 1, whereas if the enrichment is lean to the downregulated genes, the value takes smaller than 1. Also, in the analyses for disease-specific transcriptome signatures, we selected ChIP-seq data in appropriate cell types by considering the pathogenesis of each disease (Supplementary Table 1).

In the case of analysis of drug-induced transcriptome signature, ${{{\boldsymbol{x}}}}^{{{{\mathrm{trans}}}}}$, we defined the enrichment score, $x_i^{{{{\mathrm{reg}}}}}$, for transcription factor (TF) *i* (*i* = 1, 2, …, *n*) as follows:

3 $$x_i^{{{{\mathrm{reg}}}}} = \left\{ {\begin{array}{*{20}{c}} { + \left| {{{{\mathrm{log}}}}_{10}\left( {P_i} \right)} \right|,\left( {FE \ge 1} \right),} \\ { - \left| {{{{\mathrm{log}}}}_{10}\left( {P_i} \right)} \right|,\left( {FE \,<\, 1} \right),} \end{array}} \right.$$

where *n* is the total number of TFs and ${P_{i}}$ is the FDR-corrected ${P_{i}}$-value that was calculated using Fisher’s exact test based on the occurrence probability as follows:

4 $$\psi _i = \frac{{\left( {a + b} \right)!\left( {c + d} \right)!\left( {a + c} \right)!\left( {b + d} \right)!}}{{m!a!b!c!d!}},$$

where *m* is the total number of regulated genes, *a* is the number of TF_*i*_ that binds to upregulated genes, *b* is the number of TF_*i*_ that does not bind to upregulated genes, *c* is the number of TF_*i*_ that binds to downregulated genes, and *d* is the number of TF_*i*_ that do not bind to downregulated genes. Note that positive and negative scored transcription factors were considered to be enriched around up- and downregulated genes in the transcriptome signature, respectively. For the disease-specific transcriptome signatures, ${{{\boldsymbol{y}}}}^{{{{\mathrm{trans}}}}}$, we similarly defined the enrichment scores, $y_i^{{{{\mathrm{reg}}}}}$, respectively (see Supplementary notes and Supplementary Fig. 11).

### Construction of regulome-based signatures

For drugs, we represented each drug-induced regulome signature using a feature vector as follows:

5 $${{{\boldsymbol{x}}}}^{{{{\mathrm{reg}}}}} = \left( {x_1^{{{{\mathrm{reg}}}}},x_2^{{{{\mathrm{reg}}}}}, \cdots ,x_s^{{{{\mathrm{reg}}}}}} \right)^{{{\mathrm{T}}}},$$

where *s* is the number of transcription factors (*s* is 582 in this study). For diseases, we represented each disease-specific regulome signature using a feature vector as follows:

6 $${{{\boldsymbol{y}}}}^{{{{\mathrm{reg}}}}} = \left( {y_1^{{{{\mathrm{reg}}}}},y_2^{{{{\mathrm{reg}}}}}, \cdots ,y_t^{{{{\mathrm{reg}}}}}} \right)^{{{\mathrm{T}}}},$$

where *t* is the number of transcription factors (*t* is 711 in this study). 573 of 711 transcription factors were in common with transcription factors in drug-induced regulome signature, ${{{\boldsymbol{x}}}}^{{{{\mathrm{reg}}}}}$. Each element of regulome signatures is the enrichment score for a transcription factor.

### Prediction of drug indications

In general, drug-induced transcriptome signatures are assumed to be inversely correlated with disease-specific transcriptome signatures if the drugs have therapeutic effects on the corresponding diseases^12^. Therefore, the inverse correlation method is a popular transcriptome-based drug repositioning approach to find new drugs for diseases^10,21–23^. Accordingly, in the transcriptome-based prediction of drug indications, we defined the prediction score for diseases as follows:

7 $$S^{{{{\mathrm{trans}}}}}\left( {{{{\boldsymbol{x}}}}^{{{{\mathrm{trans}}}}},{{{\boldsymbol{y}}}}^{{{{\mathrm{trans}}}}}} \right) = - {{{\mathrm{cos}}}}\left( {{{{\boldsymbol{x}}}}^{{{{\mathrm{trans}}}}},{{{\boldsymbol{y}}}}^{{{{\mathrm{trans}}}}}} \right),$$

where ${{{\mathrm{cos}}}}\left( \cdot \right)$ is a function of cosine correlation. A drug that has a high *S*^trans^ was assumed to be a candidate for treating diseases.

In contrast, in the regulome-based prediction of drug indications, the inverse correlation method is expected not to work well due to the observation of positive correlations between diseases and their approved drugs (Fig. 3b). Therefore, we defined the prediction score for diseases as follows:

8 $$S^{{{{\mathrm{reg}}}}}\left( {{{{\boldsymbol{x}}}}^{{{{\mathrm{reg}}}}},{{{\boldsymbol{y}}}}^{{{{\mathrm{reg}}}}}} \right) = \left\{ {\begin{array}{*{20}{c}} {\mathrm{cos}\left( {{{{\boldsymbol{x}}}}^{{{{\mathrm{reg}}}}},{{{\boldsymbol{y}}}}^{{{{\mathrm{reg}}}}}} \right),\left( {\overline {{{{\mathrm{cos}}}}\left( {{{{\boldsymbol{x}}}}_{{{{\mathrm{approved}}}}}^{{{{\mathrm{reg}}}}},{{{\boldsymbol{y}}}}^{{{{\mathrm{reg}}}}}} \right)} \ge 0.0} \right),} \\ { - \mathrm{cos}\left( {{{{\boldsymbol{x}}}}^{{{{\mathrm{reg}}}}},{{{\boldsymbol{y}}}}^{{{{\mathrm{reg}}}}}} \right),\left( {\overline {{{{\mathrm{cos}}}}\left( {{{{\boldsymbol{x}}}}_{{{{\mathrm{approved}}}}}^{{{{\mathrm{reg}}}}},{{{\boldsymbol{y}}}}^{{{{\mathrm{reg}}}}}} \right)} \,<\, 0.0} \right),} \end{array}} \right.$$

where ${{{\mathrm{cos}}}}\left( \cdot \right)$ is a function of cosine correlation, ${{{\boldsymbol{x}}}}_{{{{\mathrm{approved}}}}}^{{{{\mathrm{reg}}}}}$ is the regulome signature of approved drugs for the disease, and $\overline {{{{\mathrm{cos}}}}\left( \cdot \right)}$ is the median of cosine correlations. A drug that has a high *S*^reg^ was assumed to be a candidate for treating diseases.

### Drugs for experimental validation

Norethindrone (N0449), flucytosine (F0321), spironolactone (S0260), and Danazol (17230-88-5), were obtained from all Nacalai Tesque (Kyoto, Japan). All chemicals were dissolved in dimethyl sulfoxide (DMSO).

### Cell culture

Human nonsmall cell lung cancer cell lines: H1975, PC9, H3122, and HC827 were purchased from ATCC (Manassas, VA, USA), and cultured in RPMI-1640 containing 10% FBS (Thermo Fisher Scientific, Waltham, MA, USA).

### Viability and apoptosis assays

Cell viability and caspase-3/7 activities were evaluated using the CellTiter-Glo® 2.0 Cell Viability Assay kit (Promega, Madison, WI, USA) and Caspase-Glo® 3/7 3D Assay kit (Promega, Madison, WI, USA), respectively, according to the manufacturer’s instructions. H1975, PC9, H3122, and HC827 cells were plated at a density of 1.0 × 10^4^ cells/well in 50 µL of complete culture medium into 96-well plates. Then, 50 µL of each drug was added at the indicated final concentration (i.e., 0.1, 1.0, 10, and 100 µM). After 6 h, 100 µL of the Viability reagent were added to all wells. The samples were then mixed with an orbital shaker for 2 minutes and incubated at room temperature for 10 min. Luminescence was measured for viability using a plate reader (Enspire; PerkinElmer, Waltham, MA, USA). Next, H1975, PC9, H3122, and HC827 cells were plated at a density of 5.0 × 10^3^ cells/well in 10 µL of complete culture medium into 384-well plates. Then, 10 µL of each of the drugs were added at the specified final concentration. After 6 h, 20 µL of the Viability reagent were added to all wells. The samples were then mixed with an orbital shaker at 500 rpm for 30 s, and incubated at room temperature for 30 min. The caspase activation was determined by measuring the luminescence with an Enspire instrument.

### Reporting summary

Further information on research design is available in the Nature Research Reporting Summary linked to this article.

## Supplementary information

Supplementary information

Reporting Summary

## Data Availability

The datasets used and/or analyzed during the current study are available from the corresponding author on reasonable request.
