# Supplementary material for: Regulome-based characterization of drug activity across the human diseasome
Source: NPJ Syst Biol Appl. 2022 Nov 7;8:44. doi: 10.1038/s41540-022-00255-4 (PMC9640590; doi:10.1038/s41540-022-00255-4)
Supplement: Supplementary file 1 — Supplementary information [file 41540_2022_255_MOESM1_ESM.pdf]

Supplementary Information for

Regulome-based characterization of drug activity across the human diseasome

This PDF file includes:

Supplementary notes
Supplementary Tables 1 and 2
Supplementary Figures 1 to 11

Supplementary notes

Performance evaluation of the transcriptome-based method using the distinction between positive and negative correlations

We performed an additional experiment to apply a similar correction to the conventional transcriptome-based method, where positive and negative correlations were considered for calculating the prediction scores. **Supplementary Fig. 8** shows the distribution of the prediction scores for approved and other drugs by the modified transcriptome-based method with a distinction between positive and negative correlations. Although no significant difference was observed in the prediction scores between approved and other drugs (P = 0.341; Wilcoxon signed-rank test), approved drugs tend to have higher scores than other drugs. This result implies that using the distinction between positive and negative correlations could improve the performance of the conventional transcriptome-based method to some extent; however, the applicability is limited.

Performance evaluation of the transcriptome-based method with all genes

We performed an additional experiment to use all genes to construct transcriptome signatures and calculate transcriptome-based correlations. **Supplementary Fig. 9** depicts the distribution of the prediction scores for approved drugs and other drugs by

the modified transcriptome-based method with all genes. The distribution of the adjusted transcriptome-based prediction scores with all genes is quite similar to that of the original method with only regulated genes (**Fig. 4B**). No significant difference was observed in the prediction scores between approved drugs and other drugs (P = 0.711; Wilcoxon signed-rank test). This result suggests that selecting all genes does not enhance the transcriptome-based method's performance.

Anticancer activity of a compound with a low prediction score was not observed in vitro experiments

We have performed additional experiments to show that a compound with a low regulome-based prediction score causes little to no cell death. First, we selected a commercially available drug from the low-scoring drugs: etofylline (vasodilator). The prediction score was 0.142. Next, we evaluated the anticancer activity regarding cell viability. We used human non-small cell lung cancer cell lines: PC9 and H1975. Therefore, we observed no anticancer activities of etofylline (**Supplementary Fig. 10**). This result implies that a compound with a low regulome prediction score does not have anticancer activity.

Enrichment significance with varying percentages of genes

We performed additional experiments to assess the sensitivity of the entire analysis to a threshold parameter for selecting regulated genes in the regulome-based method. We selected the top/bottom 10% genes from transcriptome-based signatures as regulated genes and evaluated the enrichment of transcription factors around the regulated genes. **Supplementary Fig. 11** shows the histogram of the regulome-based correlation coefficients between the top/bottom 5% genes and the top/bottom 10% genes. The enrichment significance was calculated using Fisher's exact test. The median of correlation coefficients is 0.800. This result implies that enriched patterns of transcription factors are highly correlated between different percentages of genes.

Supplementary Table 1 | List of diseases downloaded from the CREEDS database and their cell types assigned for selecting disease-related ChIP-seq experiments.

Disease name	Cell type
Acute myeloid leukemia	Blood
Adrenoleukodystrophy	All cell types
Adrenoleukodystrophy, neonatal	All cell types
Adult T-cell leukemia	Blood
Allergic contact dermatitis	Epidermis
Alpers syndrome	All cell types
Alpha-1-antitrypsin (A1AT) deficiency	Blood
Alzheimer's disease	Neural
Amyotrophic lateral sclerosis	Neural
Aplastic anemia	Blood
Asthma	Lung
Atopic dermatitis	Blood
Breast cancer	Breast
Cerebral palsy	Blood
Cervical cancer	Uterus
Chronic granulomatous disease	Blood
Chronic lymphocytic leukemia	Blood
Chronic myeloid leukemia	Blood
Colorectal cancer	Digestive tract
Congenital muscular dystrophies	All cell types
Crohn's disease	Blood
Cystic fibrosis	All cell types
Dengue	Blood
Diamond-Blackfan anemia	Blood
Dilated cardiomyopathy	Cardiovascular
Distal myopathy	All cell types
Dystrophinopathies	All cell types
Ebola haemorrhagic fever	Blood
Endometrial cancer	Uterus
Epidermolysis bullosa simplex	All cell types
Facioscapulohumeral muscular dystrophy	All cell types
Familial combined hyperlipidemia	Blood
Fragile X syndrome	All cell types
Gastric cancer	Digestive tract
Glycerol kinase deficiency	All cell types
Hepatitis C	Liver
Huntington's disease	Neural
Hypercholesterolemia	Blood
Idiopathic pulmonary fibrosis	Lung
Immune thrombocytopenia	Blood
Inclusion body myopathy 3	All cell types
Inflammatory bowel disease	Blood
Ketosis-prone diabetes mellitus	Blood
LDL Receptor Disorder	Blood
Left ventricular noncompaction	Cardiovascular
Lewy body dementia	Neural
Malignant melanoma	Epidermis
Marfan syndrome	All cell types
MELAS Syndrome	All cell types
Mitochondrial respiratory chain deficiencies	All cell types
Mosquito-borne viral fevers	Blood
Multiple myeloma	Blood
Myotonic dystrophy	All cell types
Nasopharyngeal cancer	All cell types
Non-syndromic X-linked mental retardation	All cell types
Nonaka distal myopathy	All cell types
Ovarian cancer	Gonad
Pancreatic cancer	Pancreas
Parkinson's disease	Neural
Peroxisome biogenesis disorder	All cell types
Pituitary adenomas	All cell types
POLG related disorders	All cell types
Primary dystonia	All cell types
Primary open angle glaucoma	All cell types
Renal cell carcinoma	Kidney
Rett syndrome	Neural
Rheumatoid arthritis	Blood
Sarcoidosis, early-onset	Blood
Severe acute respiratory syndrome	Blood
Sickle cell anemia	Blood
Small cell lung cancer	Lung
Systemic lupus erythematosus	Blood
Testicular cancer	Gonad
Tuberculosis	Lung
Type I diabetes mellitus	Blood
Type II diabetes mellitus	Pancreas
Ulcerative colitis	Blood
Williams-Beuren syndrome	All cell types
46,XY disorders of sex development	All cell types

Supplementary Table 2 | Statistics of TCGA transcriptome data (downloaded on Feb. 22th, 2021).

Study abbreviation	Study name	Number of cases	Number of samples	Sample type (code)		
				Primary Solid Tumor (01)	Recurrent Solid Tumor (02)	Solid Tissue Normal (11)
LUAD	Lung adenocarcinoma	515	594	533	2	59
LUSC	Lung squamous cell carcinoma	501	551	502	-	49

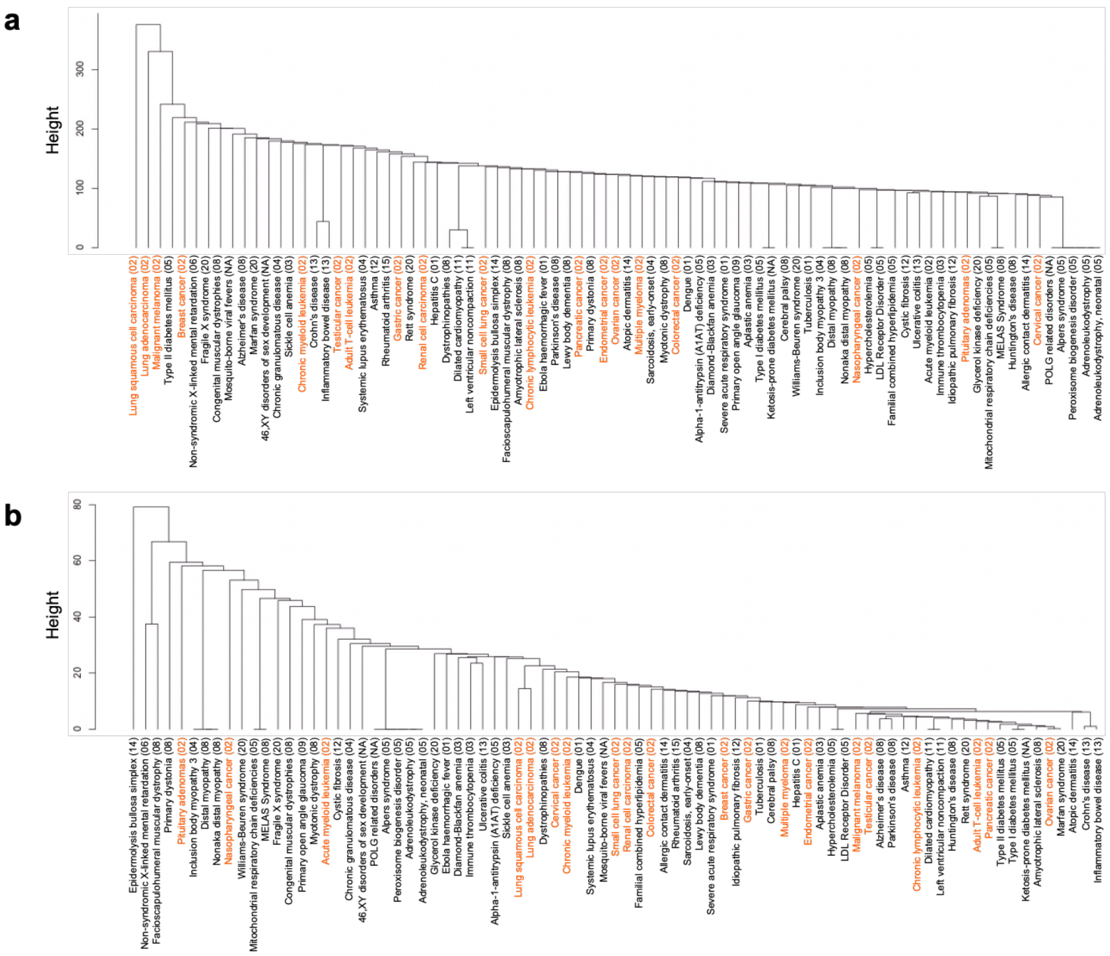

Supplementary Figure 1 | The clustering of diseases based on **(a)** transcriptome signatures and **(b)** regulome signatures. Each dendrogram was constructed using the “average linkage” algorithm. The number in the bracket shows the ICD-11 disease chapter. Diseases belonging to chapter 02 (neoplasms) are highlighted.

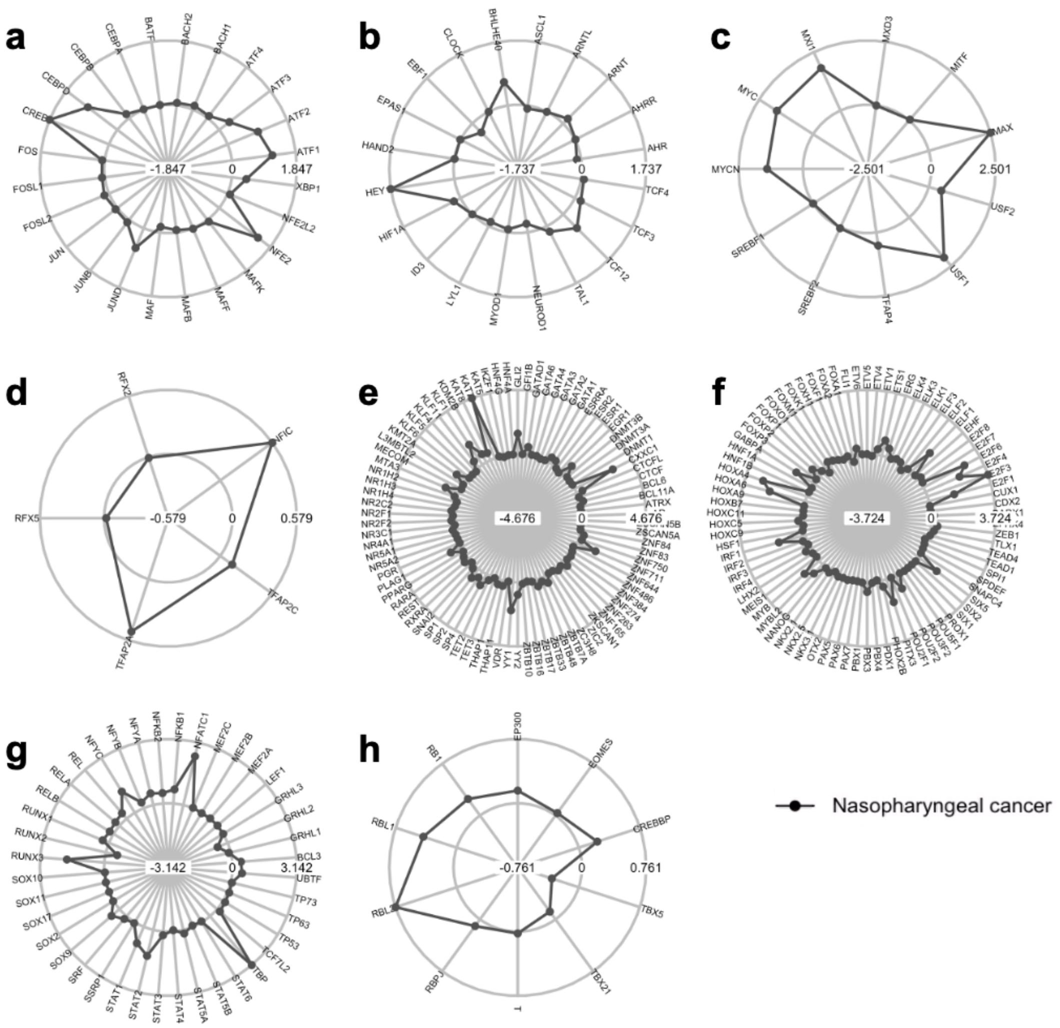

Supplementary Figure 2 | Radial plot of enrichment scores for transcription factors in the regulome signature of nasopharyngeal cancer. Transcription factors are shown for each category: **(a)** Basic leucine zipper (bZIP), **(b)** Basic helix-loop-helix (bHLH), **(c)** Basic helix-loop-helix leucine zipper (bHLH-ZIP), **(d)** Other basic domains, **(e)** Zinc finger, **(f)** Helix-turn-helix, **(g)** beta-Scaffold factors with minor groove contacts, and **(h)** Other transcription factors.

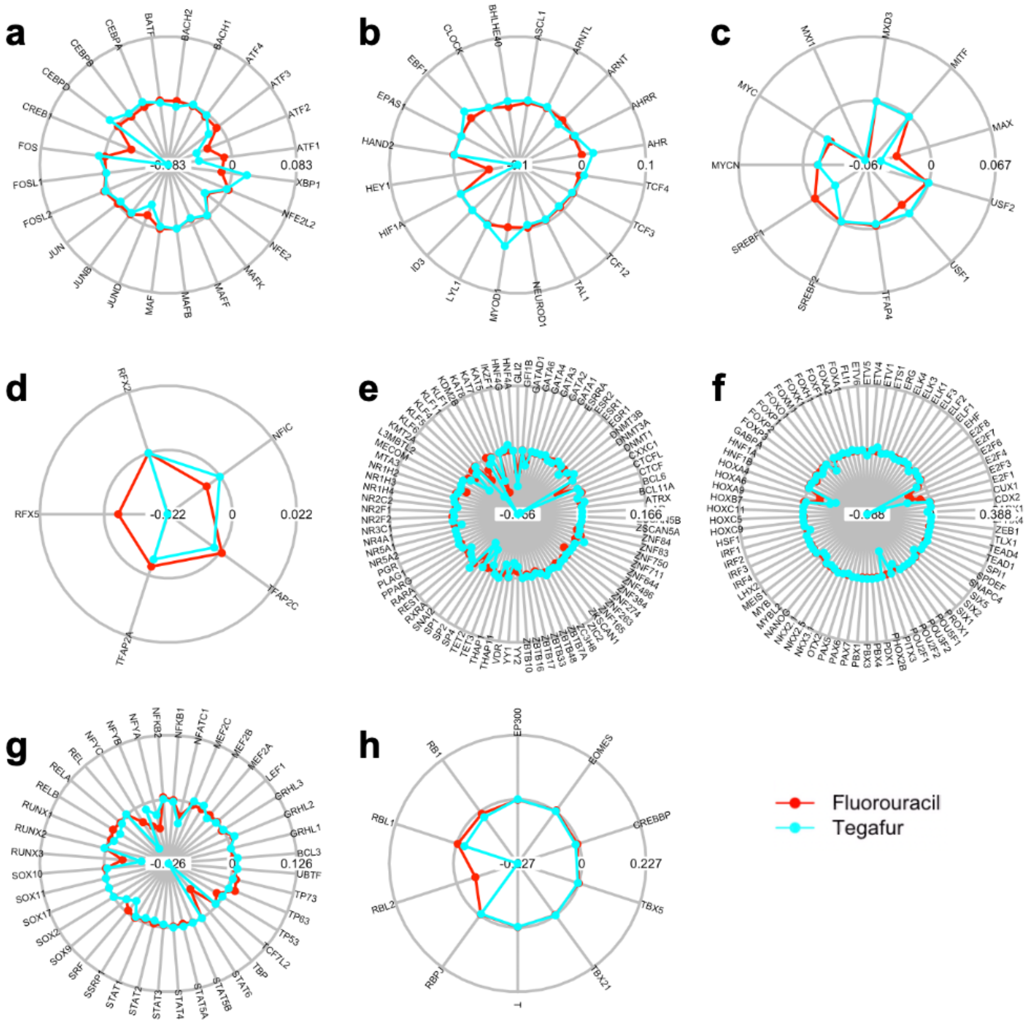

Supplementary Figure 3 | Radial plot of enrichment scores for transcription factors in the regulome signature of approved drugs for nasopharyngeal cancer. Transcription factors are shown for each category: **(a)** Basic leucine zipper (bZIP), **(b)** Basic helix-loop-helix (bHLH), **(c)** Basic helix-loop-helix leucine zipper (bHLH-ZIP), **(d)** Other basic domains, **(e)** Zinc finger, **(f)** Helix-turn-helix, **(g)** beta-Scaffold factors with minor groove contacts, and **(h)** Other transcription factors.

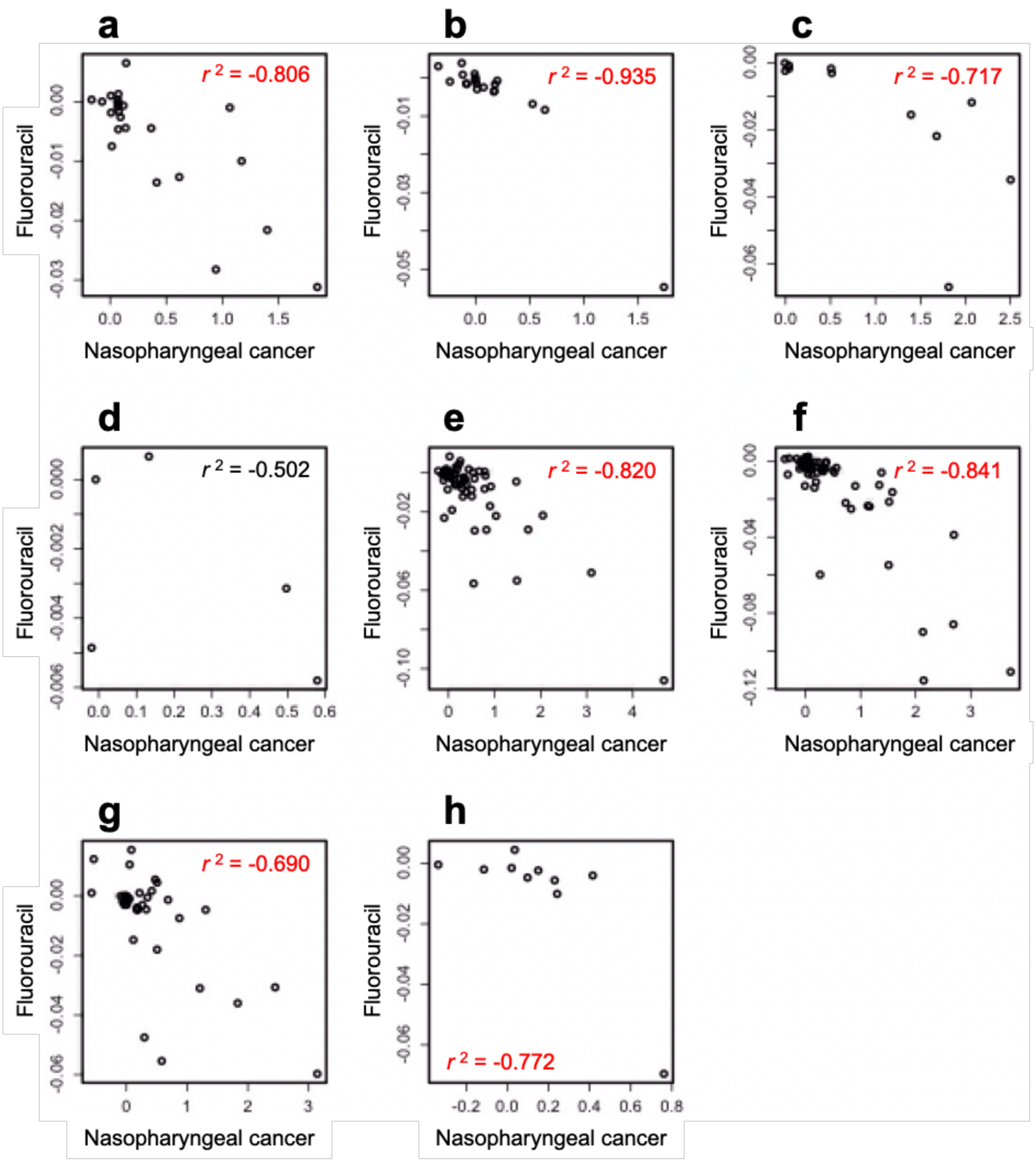

Supplementary Figure 4 | Scatter-plot of enrichment scores for transcription factors in the regulome signature of nasopharyngeal cancer and that of fluorouracil, an approved drug for nasopharyngeal cancer. Significant correlation coefficients (P < 0.05) are colored in red. Scatter plots are shown for each category of transcription factors: **(a)** Basic leucine zipper (bZIP), **(b)** Basic helix-loop-helix (bHLH), **(c)** Basic

helix-loop-helix leucine zipper (bHLH-ZIP), **(d)** Other basic domains, **(e)** Zinc finger, **(f)** Helix-turn-helix, **(g)** beta-Scaffold factors with minor groove contacts, and **(h)** Other transcription factors.

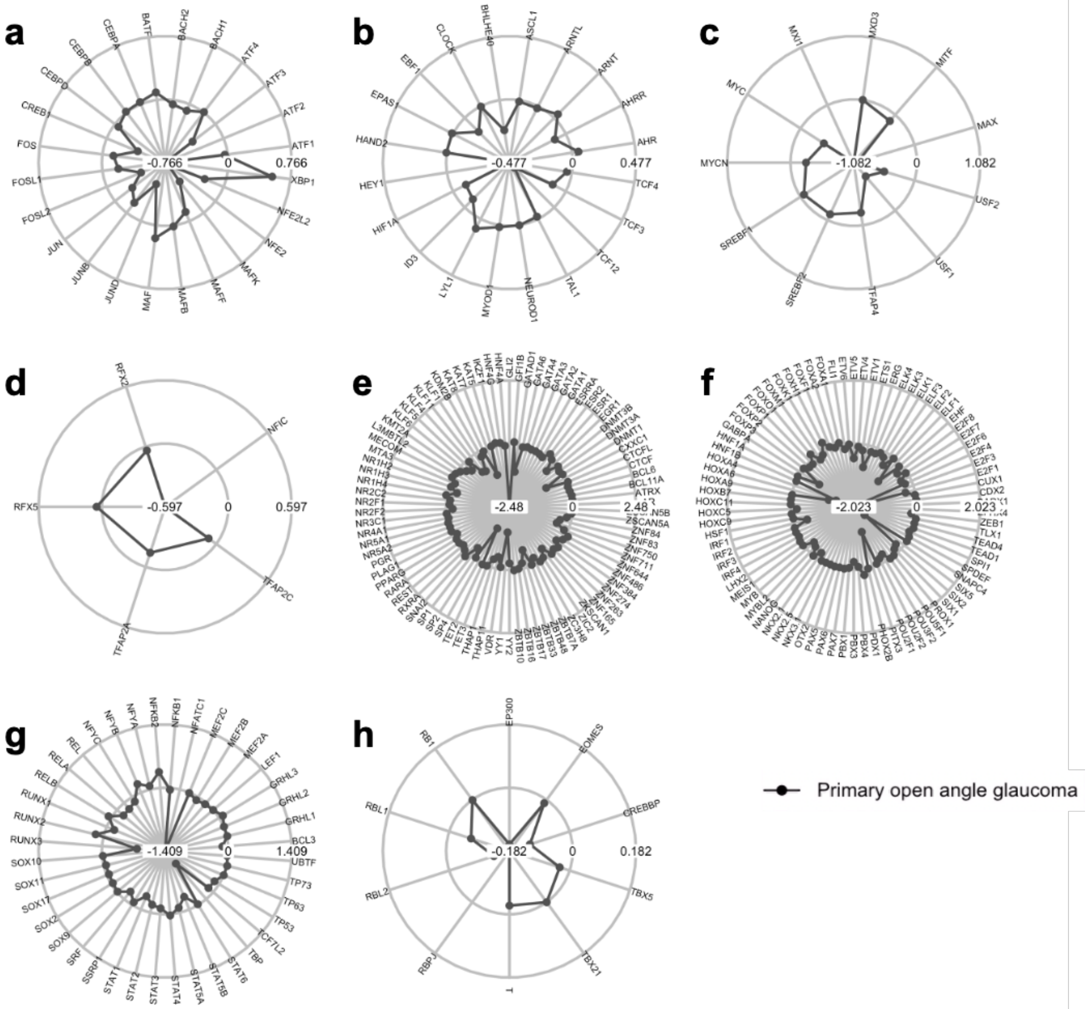

Supplementary Figure 5 | Radial plot of enrichment scores for transcription factors in the regulome signature of primary open angle glaucoma. Transcription factors are shown for each category: **(a)** Basic leucine zipper (bZIP), **(b)** Basic helix-loop-helix (bHLH), **(c)** Basic helix-loop-helix leucine zipper (bHLH-ZIP), **(d)** Other basic domains, **(e)** Zinc finger, **(f)** Helix-turn-helix, **(g)** beta-Scaffold factors with minor groove contacts, and **(h)** Other transcription factors.

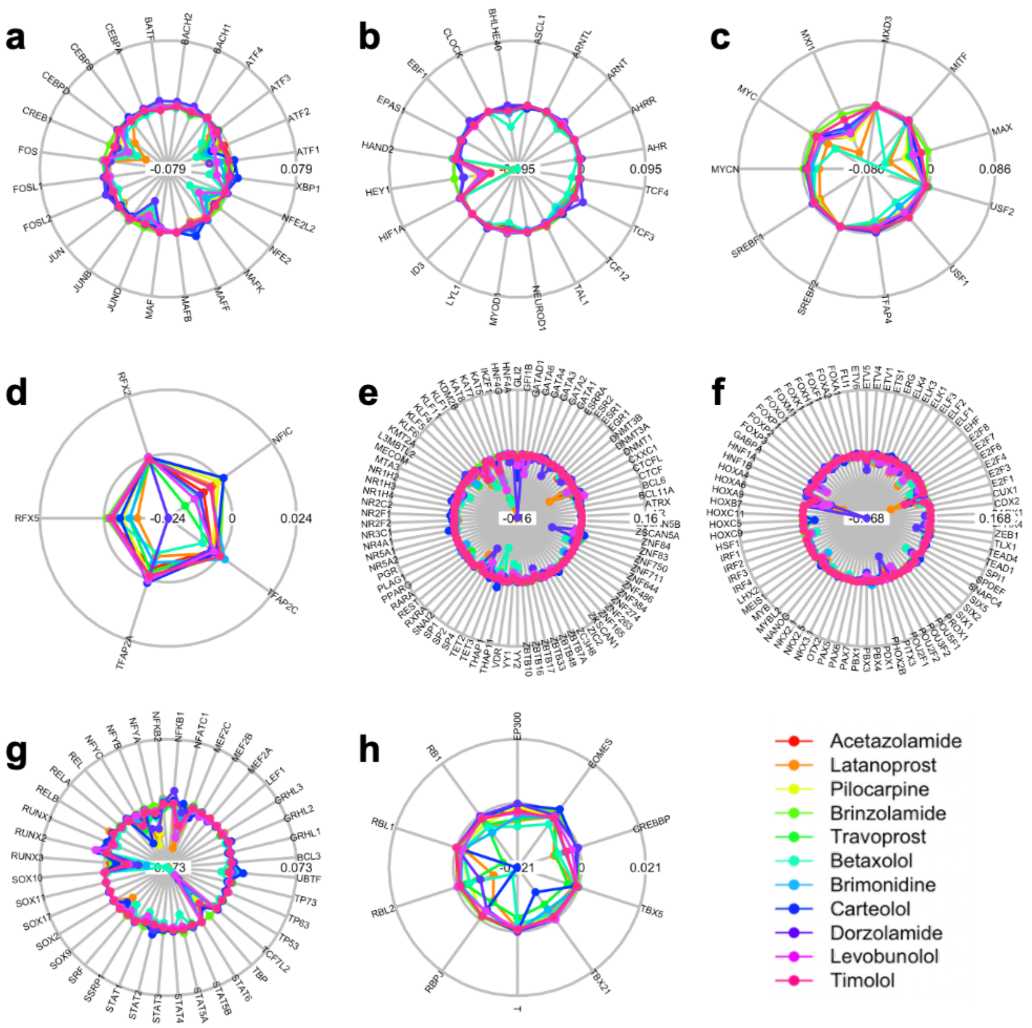

Supplementary Figure 6 | Radial plot of enrichment scores for transcription factors in the regulome signature of approved drugs for primary open angle glaucoma. Transcription factors are shown for each category: **(a)** Basic leucine zipper (bZIP), **(b)** Basic helix-loop-helix (bHLH), **(c)** Basic helix-loop-helix leucine zipper (bHLH-ZIP), **(d)** Other basic domains, **(e)** Zinc finger, **(f)** Helix-turn-helix, **(g)** beta-Scaffold factors with minor groove contacts, and **(h)** Other transcription factors.

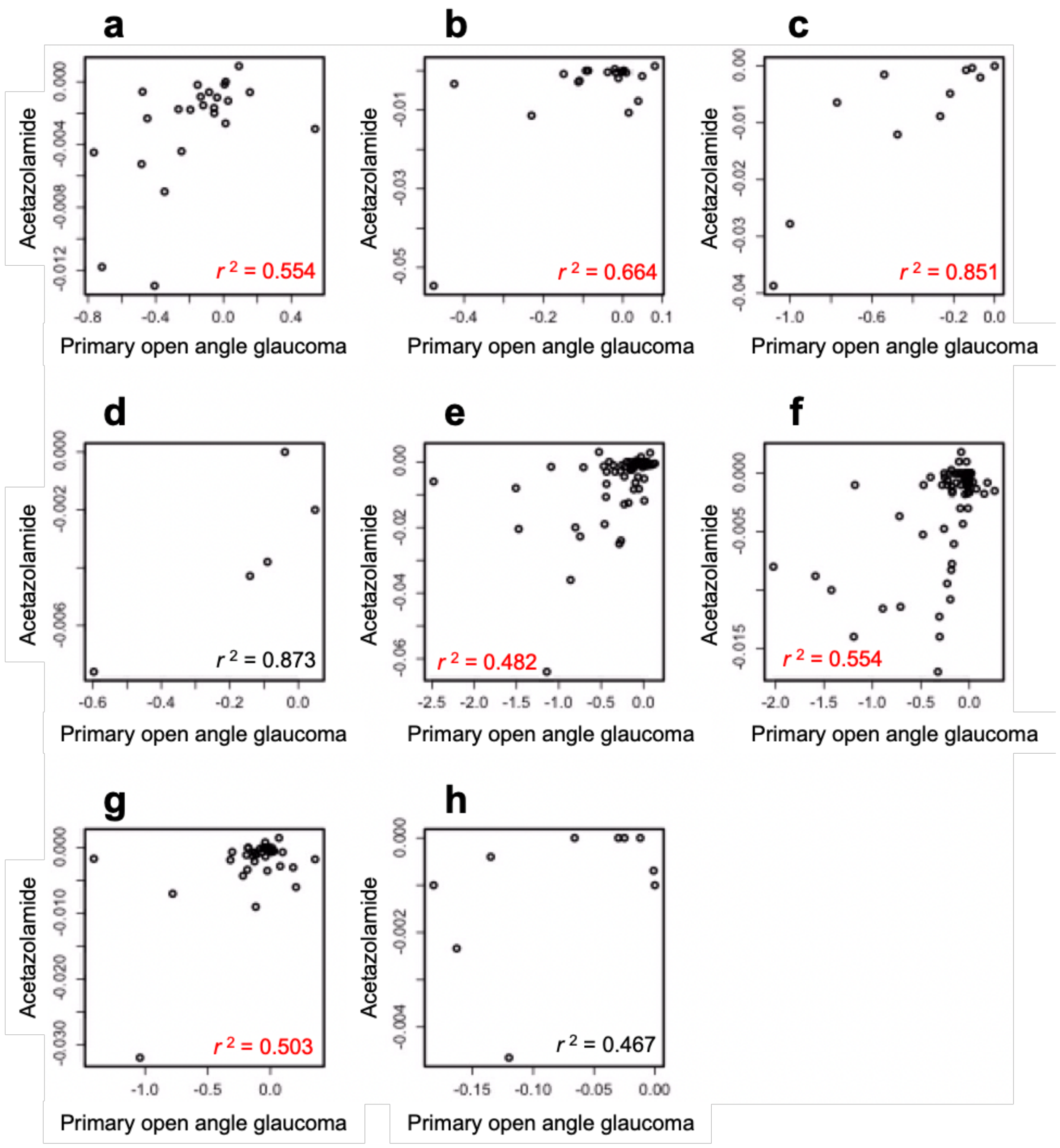

Supplementary Figure 7 | Scatter-plot of enrichment scores for transcription factors in the regulome signature of primary open-angle glaucoma and that of an approved drug, acetazolamide. Significant correlation coefficients ($P < 0.05$) are colored in red. Scatter plots are shown for each category of transcription factors: **(a)** Basic leucine zipper (bZIP), **(b)** Basic helix-loop-helix (bHLH), **(c)** Basic helix-loop-helix leucine zipper

(bHLH-ZIP), **(d)** Other basic domains, **(e)** Zinc finger, **(f)** Helix-turn-helix, **(g)** beta-Scaffold factors with minor groove contacts, and **(h)** Other transcription factors.

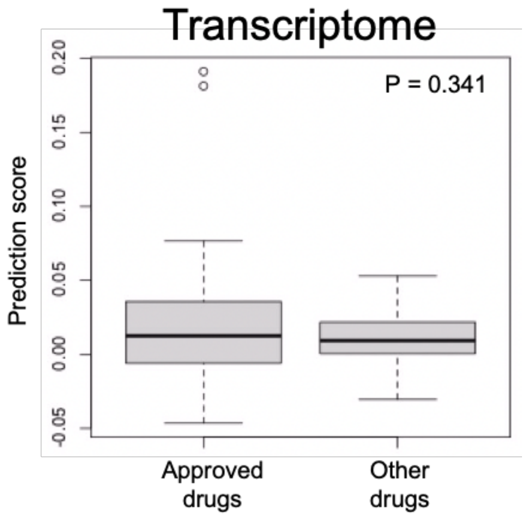

Supplementary Figure 8 | Distribution of the prediction scores for approved and other drugs by the revised transcriptome-based method, where positive and negative correlations were considered for calculating the prediction scores. P-value was determined by the Wilcoxon signed-rank test. In the box plots: center line, median; box, interquartile range; whiskers, 1.5 × interquartile range; dots, outliers.

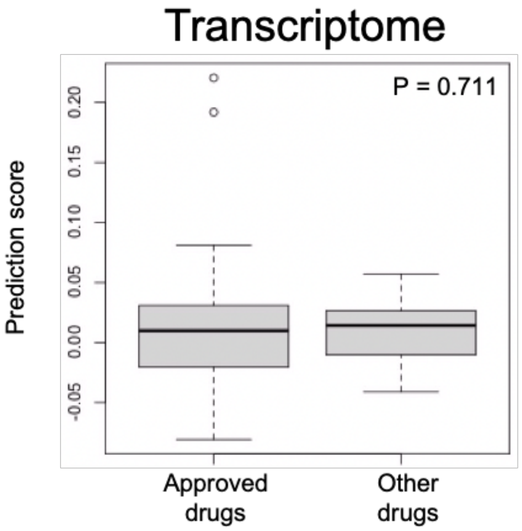

Supplementary Figure 9 | Distribution of the prediction scores for approved drugs and other drugs by the revised transcriptome-based method with all genes, where all genes in transcriptome signatures were used for calculating the regulome-based correlations. *P*-value was determined using the Wilcoxon signed-rank test. In the box plots: center line, median; box, interquartile range; whiskers, 1.5 × interquartile range; dots, outliers.

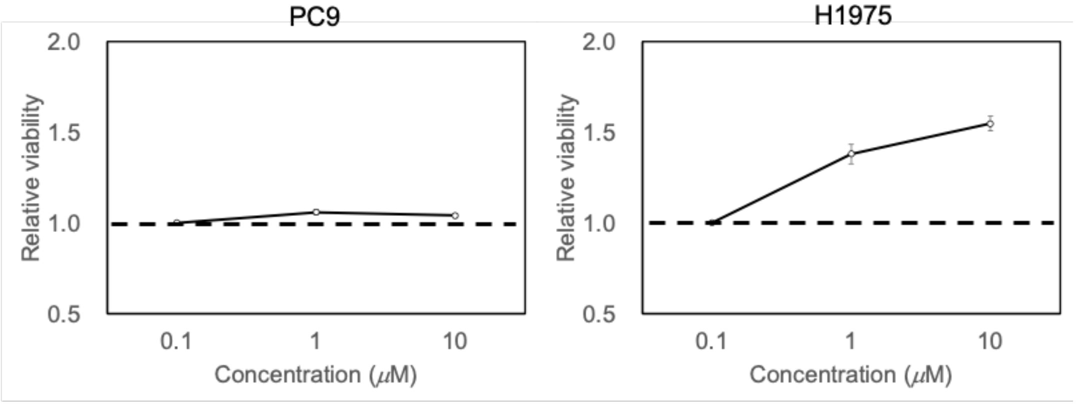

Supplementary Figure 10 | Experimental validation of little anticancer effects of etofylline, a vasodilator drug. On a logarithmic scale, the horizontal axis shows concentration. The relative viability is shown on the vertical axis. The graph depicts the average and standard deviations for three replicate studies.

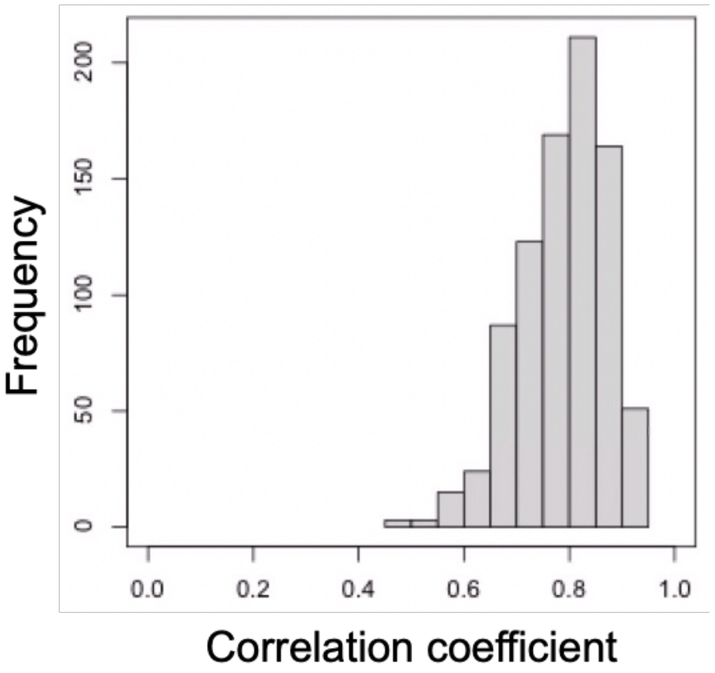

Supplementary Figure 11 | Histogram of the regulome-based correlation coefficients between the top/bottom 5% genes and the top/bottom 10% genes for randomly selected drugs, where the enrichment significance was calculated using Fisher's exact test.
