# Supplementary material for: Regulome-based characterization of drug activity across the human diseasome
Source: NPJ Syst Biol Appl. 2022 Nov 7;8:44. doi: 10.1038/s41540-022-00255-4 (PMC9640590; doi:10.1038/s41540-022-00255-4)
Supplement: Supplementary file 2 — Reporting Summary [file 41540_2022_255_MOESM2_ESM.pdf]

nature portfolio

Corresponding author(s): Yoshihiro Yamanishi

Last updated by author(s): Oct 13, 2022

Reporting Summary

Nature Portfolio wishes to improve the reproducibility of the work that we publish. This form provides structure for consistency and transparency in reporting. For further information on Nature Portfolio policies, see our Editorial Policies and the Editorial Policy Checklist.

Statistics

For all statistical analyses, confirm that the following items are present in the figure legend, table legend, main text, or Methods section.

n/a	Confirmed	
☐	☒	The exact sample size (*n*) for each experimental group/condition, given as a discrete number and unit of measurement
☐	☒	A statement on whether measurements were taken from distinct samples or whether the same sample was measured repeatedly
☐	☒	The statistical test(s) used AND whether they are one- or two-sided<br>*Only common tests should be described solely by name; describe more complex techniques in the Methods section.*
☐	☒	A description of all covariates tested
☐	☒	A description of any assumptions or corrections, such as tests of normality and adjustment for multiple comparisons
☐	☒	A full description of the statistical parameters including central tendency (e.g. means) or other basic estimates (e.g. regression coefficient) AND variation (e.g. standard deviation) or associated estimates of uncertainty (e.g. confidence intervals)
☐	☒	For null hypothesis testing, the test statistic (e.g. *F*, *t*, *r*) with confidence intervals, effect sizes, degrees of freedom and *P* value noted<br>*Give P values as exact values whenever suitable.*
☒	☐	For Bayesian analysis, information on the choice of priors and Markov chain Monte Carlo settings
☒	☐	For hierarchical and complex designs, identification of the appropriate level for tests and full reporting of outcomes
☒	☐	Estimates of effect sizes (e.g. Cohen's *d*, Pearson's *r*), indicating how they were calculated

Our web collection on statistics for biologists contains articles on many of the points above.

Software and code

Policy information about availability of computer code

Data collection	ChIP-Atlas database
Data analysis	ChIP-Atlas database

For manuscripts utilizing custom algorithms or software that are central to the research but not yet described in published literature, software must be made available to editors and reviewers. We strongly encourage code deposition in a community repository (e.g. GitHub). See the Nature Portfolio guidelines for submitting code & software for further information.

Data

Policy information about availability of data

All manuscripts must include a data availability statement. This statement should provide the following information, where applicable:

- Accession codes, unique identifiers, or web links for publicly available datasets
- A description of any restrictions on data availability
- For clinical datasets or third party data, please ensure that the statement adheres to our policy

The datasets used and/or analyzed during the current study are available from the corresponding author on reasonable request. The drug-induced transcriptome data were downloaded from the LINCS database and the NCBI GEO (accession numbers, GSE70138 and GSE92742). The disease-specific transcriptome data with patients of various diseases were obtained from the CREEDS database. Gene expression profiles with patients with nonsmall cell lung cancers; i.e., lung adenocarcinoma and lung squamous cell carcinoma, were obtained from TCGA program (https://www.cancer.gov/tcga).

Human research participants

Policy information about studies involving human research participants and Sex and Gender in Research.

Reporting on sex and gender	n/a
Population characteristics	n/a
Recruitment	n/a
Ethics oversight	n/a

Note that full information on the approval of the study protocol must also be provided in the manuscript.

Field-specific reporting

Please select the one below that is the best fit for your research. If you are not sure, read the appropriate sections before making your selection.

☒ Life sciences ☐ Behavioural & social sciences ☐ Ecological, evolutionary & environmental sciences

For a reference copy of the document with all sections, see nature.com/documents/nr-reporting-summary-flat.pdf

Life sciences study design

All studies must disclose on these points even when the disclosure is negative.

Sample size	No sample size calculation was performed for this study. For the drug-induced transcriptome data, we used 312,596 compound-treatment profiles (denoted as "trt_cp") in total. For the disease-specific transcriptome data with patients of various diseases, we extracted the profiles obtained from humans, which resulted in 79 diseases and 14,804 genes. For the disease-specific transcriptome data with nonsmall cell lung cancer, we used the profiles obtained from lung adenocarcinoma and lung squamous cell carcinoma, which resulted in 1,145 samples from 1,016 cases of 2 cancer studies in total.
Data exclusions	No data were excluded from the analyses.
Replication	We assessed our findings on different datasets. All replication attempts were successful.
Randomization	Our study is not a clinical study. Thus, no samples were allocated into experimental groups.
Blinding	Our study is not a clinical study. Thus, group allocation during data collection and analysis are out of scope.

Reporting for specific materials, systems and methods

We require information from authors about some types of materials, experimental systems and methods used in many studies. Here, indicate whether each material, system or method listed is relevant to your study. If you are not sure if a list item applies to your research, read the appropriate section before selecting a response.

Materials & experimental systems

n/a	Involved in the study	
☒	☐	Antibodies
☒	☐	Eukaryotic cell lines
☒	☐	Palaeontology and archaeology
☒	☐	Animals and other organisms
☒	☐	Clinical data
☒	☐	Dual use research of concern

Methods

n/a	Involved in the study	
☒	☐	ChIP-seq
☒	☐	Flow cytometry
☒	☐	MRI-based neuroimaging
